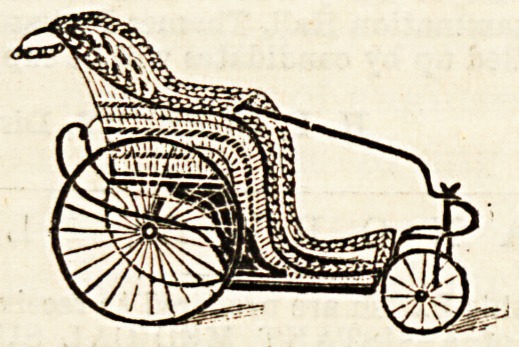# Practical Departments

**Published:** 1904-04-30

**Authors:** 


### PRACTICAL DEPARTMENTS.

### THE "RAPLIN" HAND ICE-MAKING MACHINE.

It may be said that in every institution in which is undertaken the treatment of patients, ice ready to hand is a necessity. While not much difficulty is likely to arise in the case of the larger hospitals, some of whom already possess their own refrigerating apparatus and so ensure a proper supply at an economical rate, it may sometimes happen in smaller institutions, such as cottage hospitals and nursing homes, that there is difficulty and delay in obtaining ice when it is required at short notice—for example, in the case of a head-injury. It would be well, we think, if such institutions were to supply themselves with the apparatus necessary to enable them to make their own ice as required, and so save themselves from the necessity of either continually buying ice which may not be needed, or of being stranded without any at an awkward moment. There are several forms of apparatus to be obtained which are suitable for the purpose. We have recently seen an admirable type of machine called the "Raplin," which is manufactured by the Pulsometer

Engineering Company, Limited, 61 and 63 Queen Victoria Street, London, E.C. This machine, which is worked by hand, has the great advantage of rapidity, as about half a pint of water can be frozen in five minutes. The apparatus consists of an exhaust pump, an absorber, and a carafe or cylinder which contains the water which is to be frozen.

The exhaust pump is an oil one, and is worked by turning a handle—the only manipulation which is required in making the ice. The absorber consists of a large glass vessel containing 2 quarts of commercial sulphuric acid (sp. gr. 1·846), and is connected by tubing both with the carafe and the pump. The absorber and the carafe are on a rocking-bed, which serves the double purpose of preventing the ice forming in a solid piece on the surface and obviating the formation of a layer of water on the surface of the sulphuric acid in the absorber.

The principle on which the machine works is quite simple. By means of the exhaust pump the pressure within the absorber and the vessel containing the water is reduced to such a degree that the water rapidly vaporises, while the sulphuric acid in the absorber takes up the aqueous vapour as it forms. The heat-absorption which occurs during the vaporisation is sufficient to quickly cause freezing of the remaining water. The latent heat is set free again when the vapour is taken up by the sulphuric acid, with the result that the sulphuric acid becomes hot. It is necessary to bear this in mind, as the absorber must be allowed to cool after the machine has been working for about one hour. It is obvious that the sulphuric acid will gradually become diluted until its powers of absorption are insufficient; this occurs after about 80 to 100 freezings in this climate, and the absorber must then be recharged with fresh sulphuric acid. The charging and working of the machine are quite simple if the directions supplied by the makers are faithfully observed. We might add that the "Raplin" is a very handy freezing-machine for hot climates; and its uses are not confined to making ice, as it may be employed for making ice creams, icing soda-water or champagne, and other domestic purposes. The price of the machine is £10.

### WICKER INVALID CHAIRS.

The accompanying illustration shows a wicker chair of a pattern recently supplied to a War Office order by Messrs. Stone and Sons, No. 110 in their price list. We have seen this chair at the works and found it to be of a strong, durable make, the foundation being of wood, and the back and arms only of wicker. There appears to be thus less likelihood of the floor of the chair giving way when repeatedly stepped upon by the patient, while the strain on the angle of the seat is evidently considerably less than in the case of a chair made entirely of wicker. The steel springs are leather-hung, and the cushion tyres are wired, not cemented, to the rims of the wheels. The chair is easily guided from the rear, and runs smoothly. It has a detachable steering handle, by which it can either be guided by the patient or drawn by the attendant, while another alternative is to substitute shafts for this handle, when the chair can be drawn by a donkey. The upholstering is of American leather-cloth; this, however, is a matter for individual choice, and cretonne could be used for the purpose. This firm has also just brought out a combined carrying and Merlin chair, for which they claim that it is strong, light, and that it occupies much less space than the ordinary chair, as it has no long poles at the sides, but is lifted by means of bars at the back and front. It is made on the same principle as a child's trolley, with cane seat, back, and sides, and runs very easily on small wheels. It is a neat-looking chair in light polished wood, and should prove useful where space is a consideration. The ordinary self-propelling, wicker, and bath chairs, in addition to those described above, are supplied by Messrs. Stone, whose factories and show-rooms are at Stoke Newington, London, N.

## Figures and Tables

**Figure f1:**
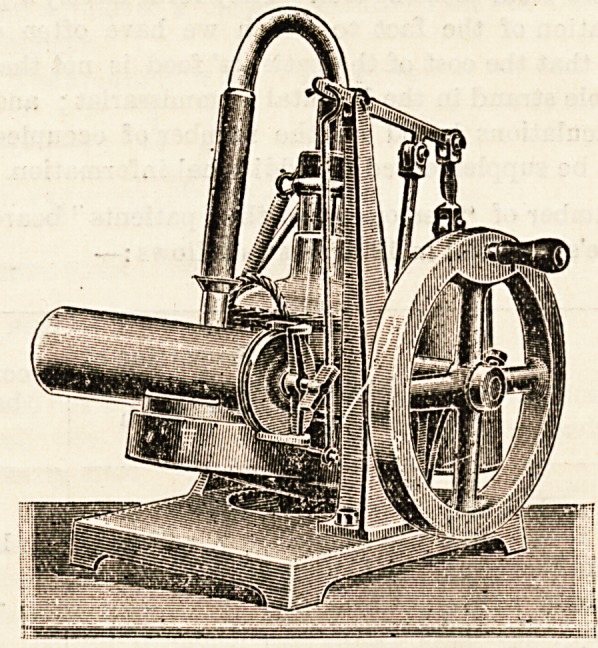


**Figure f2:**